# Self-admission to inpatient treatment in psychiatry: lessons on implementation

**DOI:** 10.1186/s12888-017-1505-x

**Published:** 2017-10-10

**Authors:** Mattias Strand, Sanna A. Gustafsson, Cynthia M. Bulik, Yvonne von Hausswolff-Juhlin

**Affiliations:** 1 Stockholm Centre for Eating Disorders, Wollmar Yxkullsgatan 27B, 118 50 Stockholm, Sweden; 2 Centre for Psychiatry Research, Department of Clinical Neuroscience, Karolinska Institutet, Stockholm, Sweden; 3 University Health Care Research Center, Faculty of Medicine and Health, Örebro University, Box 1613, 701 16 Örebro, Sweden; 4 Department of Medical Epidemiology and Biostatistics, Karolinska Institutet, 171 77 Stockholm, Sweden; 5 Department of Psychiatry, University of North Carolina at Chapel Hill, Chapel Hill, NC USA; 6 Department of Nutrition, University of North Carolina at Chapel Hill, Chapel Hill, NC USA

**Keywords:** Psychiatry, Inpatients, Patient admissions, Patient participation, Voluntary admissions

## Abstract

**Background:**

Interest has increased in programs offering self-admission to inpatient treatment for patients with severe psychiatric illness, whereby patients who are well-known to a service are afforded the opportunity to admit themselves at will for a brief period of time. The aim of the present study was to examine patient experiences of practical considerations during the start-up phase of a self-admission program in an eating disorder service.

**Methods:**

Sixteen adult participants in a self-admission program at a specialist eating disorders service were interviewed at 6 months about their experiences during the implementation phase. A qualitative content analysis approach was applied in order to identify recurring themes.

**Results:**

Six subcategories regarding implementation and logistics of self-admission were identified: “Start-up problems”, “Problems associated with reserving a bed”, “Lack of staff continuity”, “Not enough emphasis on long-term goals”, “Too demanding in terms of freedom and responsibility”, and “Suggestions for alternative models”.

**Conclusions:**

Practical recommendations can be offered for the implementation of future self-admission programs, such as thoroughly informing all participants about the rationale behind self-admission with particular emphasis on patient accountability, establishing a waiting list procedure for occasions when all designated beds are occupied, and assigning an individual contact staff member responsible for each self-admitted patient.

**Trial registration:**

The study protocol is retrospectively registered at ClinicalTrials.gov as ID: NCT02937259 .

**Electronic supplementary material:**

The online version of this article (10.1186/s12888-017-1505-x) contains supplementary material, which is available to authorized users.

## Background

Self-admission to inpatient treatment for patients with severe psychiatric illness has been offered in Norway and the Netherlands for over a decade [1], and similar pilot models have recently been introduced in Sweden [2] and Denmark [3]. In self-admission, patients who are well-known to a service are offered the possibility of self-admission at will to inpatient treatment for a brief period of time, usually 3-7 days. Patients are also free to discharge at will. Self-admission is constructed as an add-on tool rather than as a replacement of other treatment options and admission through regular procedures is still available if needed. The focus during the brief self-admission episodes is usually on milieu therapeutic effects of the hospital ward, such as increasing daily structure, resting, and reducing loneliness; in many instances, there is an explicit de-emphasis on medical interventions during self-admission and medication changes may be advised against [1].

The rationale behind self-admission is multifold, including increasing patient autonomy and agency, promoting self-monitoring and adequate help-seeking behaviors, avoiding repeated visits to the emergency ward, reinforcing the function of the inpatient ward as a safe haven, reducing coercive interventions, and reducing total time spent in inpatient treatment [1, 4, 5]. Generally, it is hypothesized that if patients can learn to identify early signs of psychiatric deterioration and act on them by admitting themselves for a brief period of time, longer regular admission episodes may be avoided. Previous research has shown promising results regarding several of these outcomes, albeit studies have mostly been small and of limited evidence level [1]. In Norwegian qualitative studies, informants have emphasized how the novel perspective offered by the self-admission approach in and of itself could transform patients’ relationship to and use of health care services [6, 7]. However, whereas previous quantitative studies have reported dramatic reductions of the total time spent in inpatient treatment [1], recent randomized controlled Norwegian studies show no differences between self-admission and treatment as usual regarding patient activation and recovery at 4 months [8] or service use at 12 months [9]; in the latter study, both groups reduced their service use equally.

Following the mostly promising results seen in research on the Norwegian self-admission programs, there is currently an intensified interest in self-admission in other Scandinavian countries [2, 3, 10]. New self-admission programs in psychiatry have been launched at several sites in both Sweden and Denmark in the past few years. However, despite this intensified interest, little is known about practical and logistical considerations in implementing new self-admission programs.

## Methods

### Aim

The aim of the present study was to examine patient experiences of practical considerations during the start-up phase of a self-admission program in an eating disorder service, in order to be able to offer advice on “dos and don’ts” in the implementation of similar programs in psychiatry.

### Setting and participants

The present study took place at the Stockholm Centre for Eating Disorders (SCÄ), which is a public specialist eating disorder service run by the Stockholm County Council. The catchment area is Metropolitan Stockholm in Sweden with a population of 2.2 million. The details of the self-admission program at SCÄ have been described elsewhere [2]. In short, since August 2014, two beds out of 11 at the adult inpatient ward have been reserved for patients with a contract for self-admission. To be eligible for the self-admission program, patients must maintain continuous treatment contact at the adult outpatient or day treatment units. They must also have had a minimum of one treatment episode in the adult inpatient ward during the past 3 years and thus be familiar with the framework and routines at the ward. Exclusion criteria are current suicidal or self-injurious behavior and/or the presence of untreated substance use. Patients may self-admit for up to 7 days at a time as often as they want by contacting the ward directly. In contrast to some other self-admission programs, there is no “quarantine period” between self-admissions during which admission is off limits. If both designated beds are occupied by other patients, a waiting list is established. Patients are offered a self-admission contract for 1 year at a time, with the possibility of annual renewal.

At the time of this study, 18 patients were enrolled in the program. All of them were asked to be interviewed about their participation, and all but two agreed. Thus, the informants in this study were 15 women and one man with a mean age of 31 years (range 18-56, median 27) enrolled in the self-admission program at SCÄ. Written informed consent was obtained. At inclusion, all informants were diagnosed with anorexia nervosa and they had been suffering from an eating disorder for a mean of 15 years (range 3-42, median 11). During their first 6 months in the program, 14 informants had made use of the opportunity to self-admit, while two of them had not; these two informants were also included in the study. Previous research has shown that participants who do not actually self-admit often find their participation meaningful nonetheless, because of increased feelings of security in knowing that the opportunity exists [1, 11]. Furthermore, when studying program implementation, it is necessary to investigate any barriers that may have kept informants from making use of it. Of those who had self-admitted, five informants had done so only once whereas two of them had made use of the opportunity 14 times (mean 5.2, median 3).

### Procedures and data analysis

All informants were interviewed about their experiences in the program at 6 months after receiving their contracts for self-admission. A semi-structured interview guide was prepared, defining the research questions that were to be investigated during the interviews (see Additional file 1 for an English translation). Some questions in the interview manual differed based on the individual informants’ use of their contract; for example, those informants who had never actually self-admitted during their time in the program where asked specific questions about the reasons behind this, whereas they could naturally not be interviewed about their experiences of the admission process, etc. All interviews were conducted face-to-face by a single interviewer (MS), who does clinical work at SCÄ but is not directly involved in the treatment at the inpatient ward. An open interview technique was applied whereby all informants were asked the same opening question (“Could you please tell me about your experiences so far in the self-admission program?”), after which the informants were asked open-ended follow-up questions appropriate to the topics brought up in the conversation (“Are there any positive aspects of self-admission?”, “Are there any negative aspects of self-admission?”, “Do you have any suggestions for changes to the program?”, etc.). The interviews lasted for 25–75 min. All interviews were tape recorded and transcribed verbatim by the interviewer. The verbatim-transcribed interviews were then analyzed using the qualitative analysis software program NVivo 11. A conventional qualitative content analysis approach was applied [12], whereby those excerpts of the interview text that emerged as meaningful in relation to the study aim were coded and labelled according to an inductive “bottom-up” principle. There were no predefined criteria (e.g., number of informant statements needed) to aid in determining what would constitute a separate category or subcategory; instead, after the initial coding had yielded a number of statement topics, meaningful clusters were identified and developed inductively by analyzing patterns and interrelations and labelled so as to reflect nuances within the data. The two authors mainly responsible for data coding (MS and SAG) then jointly reviewed these categories and grouped them into main categories and subcategories. Having settled on a data coding scheme, MS and SAG separately re-coded the interviews in a “top-down” approach to make sure that the coding scheme was accurate and reliable. An inter-rater reliability of 91% was reached at this point. Whenever MS and SAG differed in their coding, consensus was reached through discussion.

Part of the study was aimed at identifying themes that were specific for the clinical eating disorder treatment setting, such as functions and obstacles of a nature inherent to the anorexia nervosa diagnosis. These procedures and results have been reported in detail elsewhere [11]. In addition to this, the interviews yielded material of more general interest regarding implementation and logistics of the self-admission program. These data are presented in the present study. Thus, this study does not constitute a secondary analysis. Research questions on the topic of logistics and practicalities of the model were included in the interview guide from the beginning (see Additional file 1) and these data were chosen for separate presentation on the grounds that they may provide practical guidance in future implementation of self-admission programs regardless of the targeted patient group.

The study was conducted in accordance with the Helsinki declaration and it was approved by the Regional Ethical Review Board in Stockholm, Sweden (reference no. 2014/1586-31/4 and 2015/1537-32). The study protocol is registered at ClinicalTrials.gov as ID: NCT02937259.

## Results

Six subcategories regarding the overarching theme of implementation and logistics of the self-admission program were identified: “Start-up problems”, “Problems associated with reserving a bed”, “Lack of staff continuity”, “Not enough emphasis on long-term goals”, “Too demanding in terms of freedom and responsibility”, and “Suggestions for alternative models”. Informant quotes illustrating the subcategories described below are presented in Table 1.

**Table 1:** Informant quotes

Start-up problems	
a	Patient 10: *I called once and the person who answered – I’m not sure if she was part of the regular staff at the ward – said something like: ‘No, I don’t know, you’ll have to call back later’. And that could easily have made me not call back at all, because I started hesitating.*
b	Patient 9: *I was given a date and time when I should come in, and then that same day I found out that they had offered that bed to somebody else. I felt really bad, having planned it and all – yes, it was really hard. […] I think they had forgotten that she was coming in that day. But then I was offered a bed the next day, so it was sorted out.*
Problems associated with reserving a bed	
c	Patient 1: *It was so awkward, you were thinking about it every day: when is a good time, how do I know when to call? I don’t want to call every day to check if there’s a bed available because my illness doesn’t work that way.*
d	Patient 2: *Actually, once I’ve finally decided to self-admit, as of lately both beds have frequently been occupied.*
e	Patient 13: *You have to be flexible in case others are also asking for a bed. If I call now, although I really want to come in tomorrow morning so that I have time to pack and get ready mentally, they might say ‘Ok, but someone else called too so if you want the bed you’ll have to come tonight’.*
Lack of staff continuity	
f	Patient 11: *You’re assigned someone for the day and usually you find out who that is at the morning meeting. Often they just say ‘Oh, you can come to any one of us’ and that’s too intangible for me. It would make me feel safer to know that this week, Anita is your contact nurse. She doesn’t work Wednesday and Thursday, but then Lisa is your contact. […] But I know that some patients like the fact that they don’t have a designated contact person – that it makes them feel freer, that they don’t get that hospital ward feeling.*
Not enough emphasis on long-term goals	
g	Patient 10: *It’s just not possible to achieve that much in such a short amount of time. The contract is for brief admissions and changing a behavior takes time. It’s difficult to say how this could be improved.*
Too demanding in terms of freedom and responsibility	
h	Patient 1: *This thing about deciding a lot for yourself - perhaps you need to be a little careful about that because if you get to decide for yourself, very often it’ll be the illness talking. So maybe there needs to be an open dialogue so that the staff is really responsive to what’s the illness and what’s favorable in order to move ahead.*
i	Patient 2: *If only I knew that I could handle it…I do think the freedom is a large part of what’s positive about this concept.*
j	Patient 16: *I asked for this opportuniy because I realized that it could be useful to have if things didn’t work out so well. But then when it’s time to make use of it - then it’s a whole different story. Then your will power needs to be even stronger.*
Suggestions for alternative models	
k	Patient 12: *There are two beds. I’m thinking perhaps for one of them you could sort of sign up intermittently for the next 2 months, sort of ‘I wish to come in this week’.*
l	Patient 3: *I would like to know that these 2 days – or five days – every month are my days and I’m supposed to be at the ward.*
m	Patient 3: *I’ve been at the ward a whole week without actually sleeping there. So then I’m occupying a bed although I’m not really using it. That feels so wasteful – perhaps somebody else really needs the bed and I’m only there during the days. So I think there should be a slot solely for day treatment patients.*

### Start-up problems

The occurrence of start-up problems, i.e., problems related to the initial implementation of the new routines at the ward, is not surprising given that self-admission is in many ways a novel way of thinking about inpatient treatment. On this subcategory, informants mentioned that they were sometimes given contradictory information from different staff members, which risked consolidating ambivalence about admitting themselves (quote a). On at least one occasion, a self-admission bed was “double-booked” because of misunderstanding among staff members (quote b).

### Problems associated with reserving a bed

The initial procedure whereby program participants themselves were responsible for calling back to check if one of the beds had become available was also seen as stressful and potentially discouraging; however, this problem was solved by the establishment of a separate waiting list for the self-admission beds, whereby participants are now informed by phone when a bed is available for them (quote c). Informants generally spoke about this issue as being a thing of the past and most of these obstacles seemed to have been removed at the time of the interviews.

Three informants had experienced that both designated beds had been occupied by other participants in the program when they had called to see if they could admit themselves (quote d). In addition, two informants reported that they would sometimes feel stressed when being informed that there was a bed available but that it could not be held until the next day, meaning that they would have to come to the ward immediately if they wanted to make use of the bed (quote e).

### Lack of staff continuity

Because of the brief nature of the self-admissions and the scheduled rotation of staff members at the ward, it had not been possible to assign a single personal contact staff member who the participants could see continuously for the entirety of their stay. Lack of staff continuity was thus raised as an obstacle by some informants who had previously appreciated having a fixed treatment contact during their time at the ward and found this system too vague (quote f).

### Not enough emphasis on long-term goals

On this subcategory, one informant noted that it was simply not possible to reach any long-term treatment goals during such brief admissions (quote g).

### Too demanding in terms of freedom and responsibility

Several informants expressed that while they appreciated the agency and flexibility of the self-admission model, they were not always confident in handling this somewhat unfamiliar freedom and responsibility (quotes h and i). In hindsight, some of them felt that they had entered the program with unrealistic expectations about the concept and their own current ability to make constructive use of it (quote j).

### Suggestions for alternative models

Three informants mentioned that for them, it would be useful to be able to plan their brief admissions ahead instead of having to contact the ward on short notice each time they wish to admit themselves (quote k). Generally, these suggestions were raised in a context of discussing ambivalence and difficulties in appraising one’s own mental state and responding to signs of deterioration, where having pre-booked brief admission dates was seen as a helpful solution (quote l).

As a part of the self-admission model, there is an emphasis on flexibility whereby participants can choose to keep going to work or school during the days even when they are admitted or to spend the nights at home if they merely wish to partake in the daytime routines at the ward. One informant noted that she had felt guilty about formally occupying a bed even though she chose to sleep at home and suggested an additional day treatment self-admission model for those patients who do not need an actual bed at the ward (quote m).

## Discussion

Although patients in the SCÄ self-admission program were generally satisfied with their participation [11], they identified a number of practical and logistical issues that should be taken into account during the implementation phase and made suggestion for improvements of the model. A noteworthy finding is the delicate balance raised by the informants between the positive aspects of increased autonomy inherent to the self-admission model [11] and the difficulties in handling this freedom and responsibility. Several of the informants noted that while they appreciated self-admission as a tool that puts them in charge of their treatment, they were not always confident in their own ability to utilize this increased accountability in a constructive manner. Thus, on one hand, informants emphasized the novel aspects of self-admission (accessibility, autonomy, empowerment etc.) as crucial to the concept; on the other hand, the improvements they suggested were generally more closely aligned with traditional models of inpatient treatment and in some cases, such as the wish for more staff continuity, practically incompatible with the brief admission model. For example, as previously reported, informants expressed a need for an active discussion partner, such as an outpatient therapist, who is well-informed of the model and who can act as a sounding board in aiding participants decisions regarding when they ought to make use of the self-admission opportunity [11]. While this may be a wise choice from a pragmatic point of view, it could potentially run counter to the rationale behind self-admission, such as increasing patient agency and promoting appropriate help-seeking. In the implementation of future self-admission programs, it is probably wise to avoid a strict “all or nothing” approach regarding this conflict between positive and negative aspects of increased autonomy. It is not unreasonable for patients to experience some perplexity regarding the novelties of self-admission and pragmatism is advisable in the transition phase. Nonetheless, health care providers should be aware of the potentially conflicted experiences of increased patient autonomy and accountability and plan for addressing these issues if they arise.

The present results have potential clinical implications. Based on the informants’ experiences, several practical recommendations can be made for the implementation of future self-admission programs, bearing in mind the ambiguities raised above:

1. Ensure that all potential participants are thoroughly informed about the rationale behind self-admission and the emphasis on patient accountability in particular before they accept to participate in the program. It is also advisable to discuss expectations and worries beforehand, in order to instil a nuanced understanding of the concept.
2. All involved staff members (at the inpatient ward as well as at associated outpatient facilities) must be thoroughly informed of the self-admission routines and provided with clear written reference material in order to avoid contradictory information, which could cause confusion and strengthen patient ambivalence.
3. An explicit waiting list procedure should be established for occasions when all designated beds are occupied and an active outreach approach applied in informing the patients who are on this list, so that they know how long the current maximum queuing time is and do not have to make repeated calls to the ward.
4. The assignment of an individual contact staff member to whom each self-admitted patient can turn to should be a priority. Naturally, the brief nature of self-admission episodes in combination with regular staff rotation schedules at an inpatient ward can make it difficult to achieve full continuity, but in order to counteract any negative effects of this it should nevertheless be clear who the patient’s current individual contact person is at all times.
5. In order to avoid disappointment, ensure that patients are aware of the fact that although a central part of the self-admission concept is that they are free to admit themselves for whatever reason, the brief nature of these admissions makes it unlikely that they will be able to achieve any long-term treatment goals during any one stay. Instead, self-admissions are probably best seen as booster opportunities or brief respites at times when the risk of relapse is high.

Interestingly, only one informant described the brief nature of self-admission episodes as an obstacle, and most informants did not experience poor availablity of the self-admission beds.

Some informants suggested that it should also be possible to sign up in advance for future self-admission episodes. Notably, brief preventive admissions on a pre-planned basis have previously been evaluated for patients with borderline personality disorder with promising outcomes concerning viability and cost-effectiveness [13]. While this model may very well be worth considering in certain settings, it differs conceptually from the self-admission model described here; e.g., the aspects of promoting self-monitoring and accurate help-seeking in semi-acute situations would appear to be lost in the pre-planned model.

It should be noted that the findings in this study rely solely on patient experiences. Additionally, studying provider and clinician experiences could provide complementary aspects on the implementation of self-admission. To this date, there is only one study that specifically addresses this topic [14]. As previously reported [1], medical responsibility for patients self-admitting without being assessed by a psychiatrist or emergency doctor must be clearly outlined and routines for interventions in the event of medical complications or suicidal ideation should be established.

### Strengths and limitations

This study has a number of strengths and limitations. Whereas previous qualitative studies have reported data on patients’ general experiences of self-admission, this is the first study to provide insight into practical and logistical issues to be taken into account during the implementation phase of a self-admission program in psychiatry. Participants were interviewed during ongoing program participation, which allowed them to discuss their experiences and suggestions in a “real-time” context. However, the main interviewer is employed at SCÄ (although in a different department) which could potentially have affected participants’ responses. It should also be emphasized that the SCÄ self-admission program is exclusively targeted to patients with an eating disorder, which may affect generalizability of the findings to groups with other psychiatric illness; however, eating disorder specific aspects of informants’ experiences have previously been reported elsewhere [11] and the present data was chosen to be presented separately because of its more universal nature. In terms of severity, quality of life (QoL) in patients with eating disorders has been shown to be equally or more impaired than in those with other psychiatric diagnoses [15]. Improved QoL rather than remission of disorder-specific symptoms is usually an explicit aim in self-admission programs regardless of the targeted patient group. The transferability of study findings could thus be more closely related to level of QoL impairment than to diagnosis. However, one additional factor that may affect the ability to make constructive use of self-admission is ambivalence in help-seeking; in this regard, patients with an eating disorder may be more reluctant to self-admit than other groups [11]. Potential differences such as these need to be considered in the design of future self-admission programs.

## Conclusion

This study is the first to provide patients’ experiences of participating during the implementation phase of a self-admission program in psychiatry. While informants were generally satisfied with their participation in the program they also identified a number of practical issues that should be taken into account in order to optimize the usefulness of the model. Since there is currently an intensified interest in self-admission at other sites, a number of recommendations based on informant experiences are provided to aid in the implementation of future self-admission programs.

## Additional file

Additional file 1: Interview guide. An English transcription of the interview guide used in the study (DOCX 16 kb)

## Abbreviations

QoL: Quality of life
SCÄ: Stockholm Centre for Eating Disorders (Stockholms centrum för ätstörningar)
