# Supplementary material for: Self-admission to inpatient treatment in psychiatry: lessons on implementation
Source: BMC Psychiatry. 2017 Oct 10;17:343. doi: 10.1186/s12888-017-1505-x (PMC5634866; doi:10.1186/s12888-017-1505-x)
Supplement: Additional file 1: — Interview guide. An English transcription of the interview guide used in the study (DOCX 16 kb) [file 12888_2017_1505_MOESM1_ESM.docx]

Additional file

Interview guide

Experiences of self-admission among patients at an eating disorder specialist service

Follow-up after 6 months

**Opening question:**

”Could you please tell me about your experiences so far in the self-admission program?”

**Questions to be discussed during the interview:**

- Are there any positive aspects of self-admission?
- Are there any negative aspects of self-admission?
- Has your participation in the program affected your everyday life in any way?
- Has your participation in the program affected your eating disorder in any way?
- Has your participation in the program affected your influence over the treatment in any way?
- Do your relatives know that you have a contract for self-admission? How do you think they experience your participation in the program?
- Do you have any suggestions for changes to the program?
- Are you satisified with participating in the program?
- Would you recommend this model to other persons in a similar situation?
- What are your previous experiences of patient participation in health care?

For those who HAVE used the opportunity to self-admit:

- Has it been easy to contact the ward and to self-admit?
- Wat was/were your reason/s for self-admitting?
- How long time passed between the first thoughts of self-admitting and actually contacting the ward?
- Whose idea was it that you should contact the ward and self-admit?
- Has there been any obstacles for you when you have considered self-admitting?
- How has it worked for you to self-evaluate your need for inpatient treatment?
- Has there been other occassions when you have considered self-admitting but refrained? Why?
- Has self-admission been a supportive tool for you?
- Are there things that did not work as you had hoped during self-admission?
- Are there any ways in which self-admission could become more of a supportive tool?
- Did you maintain continuity with your everyday life by work/studies/sleeping at home etc during self-admission?

For those who HAVE NOT used the opportunity to self-admit:

- What are the reasons that you have not used the opportunity to self-admit?
- Has there been occassions when you have considered self-admitting but refrained? Why?
